# Rapid Mutation of Endogenous Zebrafish Genes Using Zinc Finger Nucleases Made by Oligomerized Pool ENgineering (OPEN)

**DOI:** 10.1371/journal.pone.0004348

**Published:** 2009-02-09

**Authors:** Jonathan E. Foley, Jing-Ruey J. Yeh, Morgan L. Maeder, Deepak Reyon, Jeffry D. Sander, Randall T. Peterson, J. Keith Joung

**Affiliations:** 1 Molecular Pathology Unit, Center for Cancer Research, and Center for Computational and Integrative Biology, Massachusetts General Hospital, Charlestown, Massachusetts, United States of America; 2 Cardiovascular Research Center, Massachusetts General Hospital, Harvard Medical School, Charlestown, Massachusetts, United States of America; 3 Broad Institute of MIT and Harvard, Cambridge, Massachusetts, United States of America; 4 Department of Genetics, Development and Cell Biology, Interdepartmental Graduate Program in Bioinformatics and Computational Biology, Iowa State University, Ames, Iowa, United States of America; 5 Department of Pathology, Harvard Medical School, Boston, Massachusetts, United States of America; University of Washington, United States of America

## Abstract

**Background:**

Customized zinc finger nucleases (**ZFN**s) form the basis of a broadly applicable tool for highly efficient genome modification. ZFNs are artificial restriction endonucleases consisting of a non-specific nuclease domain fused to a zinc finger array which can be engineered to recognize specific DNA sequences of interest. Recent proof-of-principle experiments have shown that targeted knockout mutations can be efficiently generated in endogenous zebrafish genes via non-homologous end-joining-mediated repair of ZFN-induced DNA double-stranded breaks. The Zinc Finger Consortium, a group of academic laboratories committed to the development of engineered zinc finger technology, recently described the first rapid, highly effective, and publicly available method for engineering zinc finger arrays. The Consortium has previously used this new method (known as OPEN for Oligomerized Pool ENgineering) to generate high quality ZFN pairs that function in human and plant cells.

**Methodology/Principal Findings:**

Here we show that OPEN can also be used to generate ZFNs that function efficiently in zebrafish. Using OPEN, we successfully engineered ZFN pairs for five endogenous zebrafish genes: *tfr2*, *dopamine transporter*, *telomerase*, *hif1aa,* and *gridlock*. Each of these ZFN pairs induces targeted insertions and deletions with high efficiency at its endogenous gene target in somatic zebrafish cells. In addition, these mutations are transmitted through the germline with sufficiently high frequency such that only a small number of fish need to be screened to identify founders. Finally, *in silico* analysis demonstrates that one or more potential OPEN ZFN sites can be found within the first three coding exons of more than 25,000 different endogenous zebrafish gene transcripts.

**Conclusions and Significance:**

In summary, our study nearly triples the total number of endogenous zebrafish genes successfully modified using ZFNs (from three to eight) and suggests that OPEN provides a reliable method for introducing targeted mutations in nearly any zebrafish gene of interest.

## Introduction

Engineered zinc finger nucleases (**ZFN**s) form the basis of a broadly applicable technology for highly efficient genome modification [1]–[6]. ZFNs function as dimers [7] with each monomer consisting of an engineered zinc finger array (typically composed of three or four fingers) fused to a non-specific cleavage domain from the *Fok*I endonuclease [8], [9]. Zinc finger arrays in ZFNs can be engineered to bind target DNA sequences of interest [10]–[17], thereby enabling the introduction of double-strand DNA breaks (**DSB**s) into specific genomic sequences.

ZFNs can be used to alter endogenous genes in *Drosophila* and mammalian cells with absolute efficiencies ranging from 1%–50% [18]–[26]. ZFN-induced DSBs can be repaired by non-homologous end-joining (**NHEJ**), an imperfect process which frequently results in the creation of insertions and deletions (**indels**) at the site of the break. Alternatively, repair of a ZFN-induced DSB by homologous recombination (**HR**) with an appropriately designed exogenous “donor template” (an approach known as “gene targeting”) can be used to introduce a specific mutation near the break or to insert a DNA sequence at the the break.

Recent proof-of-principle studies have shown that ZFNs can also be used to create targeted NHEJ-mediated knockout mutations in endogenous zebrafish genes. Wolfe and Lawson created ZFN-induced knockouts in the *kdr* gene [27] while Amacher and colleagues mutated the *golden* and *ntl* genes [28]. These results demonstrate that ZFNs can provide an important genetic capability previously unavailable to researchers in the zebrafish field and have created much excitement in the community.

An important question raised by these groundbreaking studies is how can the typical zebrafish researcher generate the customized ZFNs required to practice this targeted knockout technology [29]. The Wolfe and Lawson *kdr* ZFNs [27] were made using a modified version of a previously described two-stage optimization strategy [30]. This approach is very difficult for the non-specialist scientist to practice because it requires the construction and interrogation of three partially randomized zinc finger libraries and of a secondary recombinant library derived from the outputs of the initial three libraries. The *ntl* and *golden* ZFNs used by Amacher and colleagues were constructed using a proprietary engineering platform developed by Sangamo BioSciences, Inc. [28]. ZFNs made by this proprietary method can be purchased from Sigma-Aldrich but the high fee charged per ZFN pair [31] may make it difficult for most labs to purchase ZFNs for more than one or two genes of interest. A third method previously used to make ZFNs (for use in other cell types) is the “modular assembly” approach in which zinc fingers with pre-selected specificities are joined together [32]–[35]. However, a recent large-scale assessment of the modular assembly method demonstrated that it is highly inefficient with a success rate for making functional ZFN pairs that is at best ∼6% [36].

The Zinc Finger Consortium recently described the development and validation of a rapid, highly effective, and publicly available method for engineering zinc finger arrays termed OPEN (for Oligomerized Pool ENgineering) [26]. OPEN requires the construction of only a single recombinant zinc finger library (smaller than 10^6^ in size) and yields ZFNs that function with high efficiencies in human and plant cells [26]. The method accounts for the context-dependent DNA-binding activities of zinc fingers, a parameter that previous studies have suggested is important for creating arrays with high DNA-binding affinities and specificities [30], [37]–[42]. In direct comparisons, OPEN exhibited a much higher success rate for yielding functional ZFNs than the modular assembly method [26]. In indirect comparisons performed with different target sites, ZFNs made by OPEN also exhibited activities and toxicities comparable to ZFNs made by the proprietary Sangamo BioSciences approach [26].

In this study, we use a modified and more rapid version of OPEN to generate ZFNs for five endogenous zebrafish gene targets. We show that these OPEN ZFNs efficiently induce indel mutations in their respective endogenous gene targets in somatic zebrafish cells. In addition, we demonstrate germline transmission of ZFN-induced mutations for four of the five gene targets. Finally, we use *in silico* analysis to show that one or more potential OPEN ZFN target sites can be found within the first three coding exons of more than 25,000 transcripts derived from endogenous zebrafish genes. Our results demonstrate that OPEN can rapidly generate ZFNs for efficient mutation of endogenous genes in zebrafish and provide strong additional support for its use with this important model organism.

## Results

### Using OPEN to engineer zinc finger arrays for endogenous zebrafish gene targets

**Figure 1:**
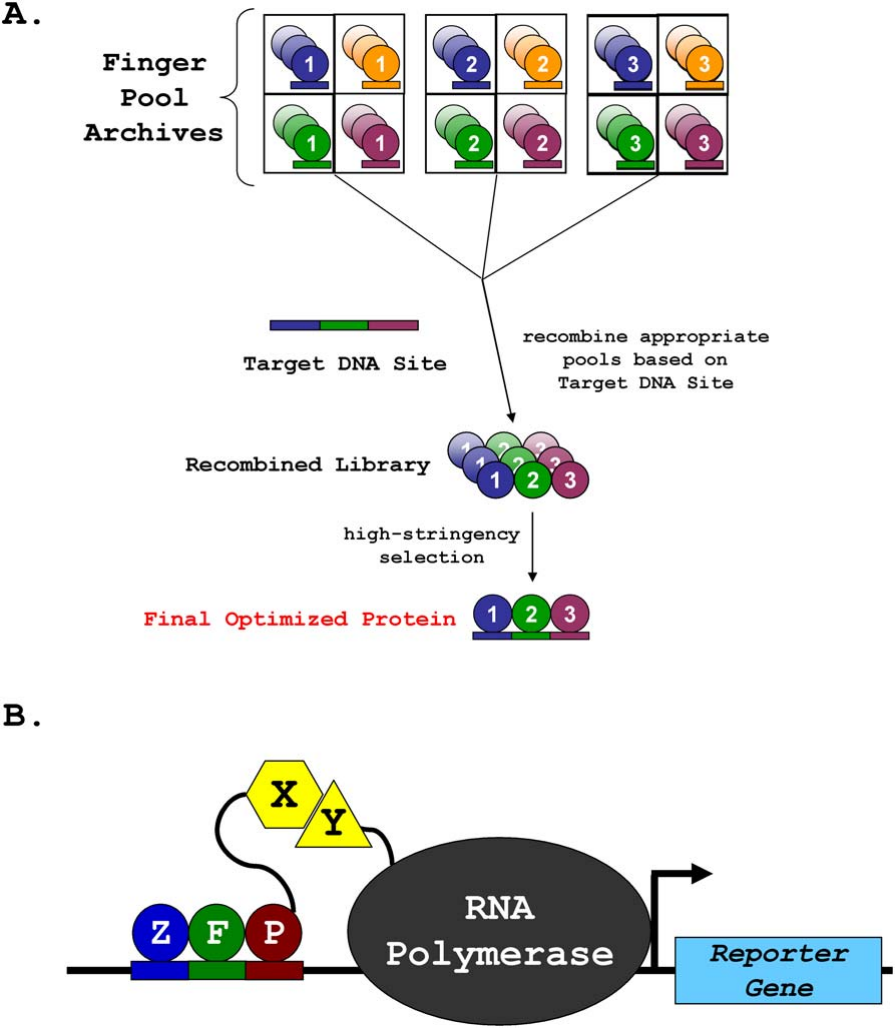
The OPEN Zinc Finger Selection Method. (A) Schematic overview of OPEN selection for a target DNA site. Zinc finger domains are shown as spheres and associated 3 bp subsites as rectangles. Details provided in the text and in Maeder et al., *Mol. Cell* 2008. (B) Schematic of the bacterial two-hybrid (B2H) system. ZFP = zinc-finger protein. X and Y = arbitrary interacting proteins.

We used the OPEN method to engineer zinc finger arrays for potential ZFN target sites in five different endogenous zebrafish genes: *dopamine transporter (dat)*, *hypoxia-inducible factor 1α* (*hif1aa*), *telomerase*, *transferrin receptor 2* (*tfr2*), and *gridlock*. The targeted genes differ widely in size, genomic location, and functional class (channel, receptor, enzyme, transcription factor) and were selected for their relevance to ongoing zebrafish research projects or for their general utility for the zebrafish community. We used the web-based ZiFiT v3.0 software program (http://bindr.gdcb.iastate.edu/ZiFiT/) [26] to identify potential target sites in the coding sequences of these genes. Ten selections (one for each half-site in the five full ZFN target sites) were performed using an improved, more rapid version of our recently described OPEN method (**Figure 1**). Alterations made to the original method included miniaturization of the selections so that they can be performed using multi-channel pipets, multi-well (24-well) blocks, and smaller amounts of solid and liquid media (see Materials and Methods). These alterations have led to a substantial increase in the speed of the procedure: as many as 48 selections can now be completed by two individuals in less than 8 weeks time.

The OPEN selections we performed successfully yielded multiple active zinc finger arrays for all 10 target half-sites (**Table 1**). As before, we defined successful arrays as those that can activate transcription of a *lacZ* reporter gene by three-fold or more in the B2H system [26]. Previous studies have shown that zinc finger arrays which activate transcription above this threshold in the B2H system possess high affinity and high specificity for their cognate DNA binding site [30]. For comparison, we also tested the zinc finger arrays from the *kdr* ZFNs previously made by Wolfe and Lawson [27] on their respective target half-sites. Interestingly, we found that although one of the arrays (ZFP1) activated transcription more than three-fold in the B2H system, the other (ZFP2) failed to show any activation (**Table 1**). The lower activity of the *kdr* ZFP2 zinc finger array in the B2H system may be due to low DNA-binding specificity as well as to the lower stringency of the bacterial one-hybrid (B1H) system used to select the *kdr*-targeted zinc finger arrays (see Discussion below).

**Table 1:** Recognition helix (RH) amino acid sequences and B2H activities of zinc finger arrays for endogenous zebrafish gene targets.

ZFN Name	Site Name	F1 subsite/RH sequence	F2 subsite/RH sequence	F3 subsite/RH sequence	Mean B2H fold-activation	S.D. of B2H fold-activation
		GTGt	GGT	GAA		
***kdr ZFP1***	kdr_2L	RSDALTR	LRHHLTR	QKANLTR	7.19	0.70
		GGAc	GTG	TTG		
***kdr ZFP2***	kdr_2R	QKGHLTR	RSDALTR	RSDSLLG	*1.14*	0.40
		GTCc	GTG	GAA		
OZ453	DT_16L	TSTLLNR	RKQVLTN	QEVNLNR	3.06	0.16
***OZ454***	DT_16L	TMAVLRR	RREVLEN	QTVNLDR	7.27	1.55
OZ455	DT_16L	TSTLLKR	RGEVLIN	QTVNLDR	2.97	0.51
OZ456	DT_16L	TRADLRR	RTEVLTN	QNTNLSR	4.35	1.45
OZ457	DT_16L	TSTLLNR	RGEVLAN	QSVNLRR	4.76	0.13
OZ458	DT_16L	TGVLLRR	RREVLMN	QDGNLGR	*1.94*	0.28
		GTCa	GAC	GGG		
OZ459	DT_16R	TRAVLAR	DAGNLTR	RIDKLGG	3.79	0.55
OZ460	DT_16R	TRAVLRR	DGSNLAR	RIDKLGG	3.80	0.02
***OZ461***	DT_16R	TAAVLTR	DRANLTR	RIDKLGD	4.18	0.05
OZ462	DT_16R	TRAVLAR	DAGNLTR	RIDKLGG	2.94	0.05
OZ463	DT_16R	TGAVLTR	DKGNLKR	RNDKLVT	3.47	0.48
		GGGt	TAG	GTG		
OZ464	HIF_12L	KAERLRR	RSDNLKS	RGDALAR	4.90	0.48
OZ465	HIF_12L	RNTHLAR	RSDNLTT	RGDALAR	8.43	0.56
OZ466	HIF_12L	KKDHLHR	RADNLQT	RKRNLTG	4.95	0.70
***OZ467***	HIF_12L	KGERLVR	RMDNLST	RKDALNR	7.39	0.51
OZ468	HIF_12L	KGERLVR	RMDNLST	RKDALNR	6.22	0.94
		GGTg	GGA	GCA		
***OZ469***	HIF_12R	IPNHLAR	QSAHLKR	QDVSLVR	7.22	0.57
OZ470	HIF_12R	IPNHLAR	QKPHLTN	QATTLRR	5.80	0.08
OZ471	HIF_12R	TKQKLQV	QNPHLTN	QSNVLSR	*0.92*	0.06
OZ472	HIF_12R	QKHHLAV	QSAHLKR	QDVSLVR	3.22	0.12
OZ473	HIF_12R	IPNHLAR	QRPHLTN	QTATLKR	3.67	0.21
		GGAg	GAT	GTA		
***OZ474***	Telo_16L	DKTKLRV	VRHNLTR	QSTSLQR	9.74	0.37
OZ475	Telo_16L	DKTKLRV	VKHNLHR	QSGTLTR	7.78	0.86
OZ476	Telo_16L	DKTKLSV	VAHNLTR	QGTSLAR	6.29	0.46
		GCTg	GAA	GAA		
OZ477	Telo_16R	QRQALDR	QTGNLLR	QRNNLGR	7.48	0.46
OZ478	Telo_16R	QRQALDR	QGSNLQR	QRNNLGR	5.79	0.17
***OZ479***	Telo_16R	SAQALAR	QGGNLTR	QHPNLTR	9.43	0.20
OZ480	Telo_16R	QRQALDR	QTGNLQR	QHPNLTR	7.72	0.67
OZ481	Telo_16R	STQALRR	QATNLQR	QHPNLTR	5.93	0.68
OZ482	Telo_16R	SRQALGR	QSANLSR	QHPNLTR	5.79	0.36
		GCTc	GGG	GGA		
OZ483	TfR2_2L	TRPMLRR	RGEHLTR	QGGHLKR	4.93	0.08
OZ484	TfR2_2L	LSQTLKR	RREHLMR	QNSHLRR	32.92	8.91
OZ485	TfR2_2L	THSMLAR	RREHLVR	QTTHLRR	5.58	0.98
OZ486	TfR2_2L	MNSTLIR	RVDHLHR	QNSHLRR	7.45	1.34
***OZ487***	TfR2_2L	MKNTLTR	RQEHLVR	QKPHLSR	7.19	0.14
OZ488	TfR2_2L	TTQALRR	RREHLMR	QTTHLSR	6.62	0.46
		GCTg	GAA	GAT		
OZ489	TfR2_2R	QRQALDR	QQTNLTR	VGGNLAR	5.52	0.66
OZ490	TfR2_2R	QRQALDR	QATNLQR	VGSNLTR	5.23	0.26
***OZ491***	TfR2_2R	SAQALAR	QQTNLAR	VGSNLTR	6.03	1.78
OZ492	TfR2_2R	QRQALDR	QSANLSR	VGSNLTR	5.63	0.42
OZ493	TfR2_2R	QRQALDR	QGGNLTR	VGGNLSR	6.57	0.12
OZ494	TfR2_2R	QRQALDR	QQTNLTR	VGSNLTR	6.76	0.75
		GGAa	GCA	GCA		
OZ495	Grck_5L	QQAHLVR	QAETLKR	QTATLKR	3.44	0.33
OZ496	Grck_5L	QQAHLVR	QAETLKR	QTATLKR	3.60	0.29
OZ497	Grck_5L	DNAHLAR	QGETLKR	QGNSLNR	*1.20*	0.15
OZ498	Grck_5L	QQAHLVR	QTETLKR	QTATLKR	3.04	0.11
***OZ499***	Grck_5L	QQAHLVR	QTETLKR	QTATLKR	3.23	0.16
OZ500	Grck_5L	QQAHLVR	QNETLRR	QTATLKR	2.82	0.14
		GAGc	GCA	GCA		
OZ501	Grck_5R	KHSNLTR	QTETLKR	QTATLKR	6.54	1.54
***OZ502***	Grck_5R	KHSNLTR	QKETLNR	QPNTLTR	10.24	0.75
OZ503	Grck_5R	KHSNLTR	QKETLNR	QPNTLTR	9.93	0.09
OZ504	Grck_5R	KHSNLTR	QMETLKR	QGGTLRR	10.98	1.62
OZ505	Grck_5R	KHSNLAR	QRETLKR	QGGTLVR	8.84	0.88
OZ506	Grck_5R	KHSNLTR	QRETLKR	QGGTLRR	7.87	2.44

Each OPEN zinc finger array was assigned an OZ___ designation which permits their unique identification in the web-based Zinc Finger Database (ZiFDB) program [47]. Previously published zinc finger arrays targeted to the *kdr* gene (isolated by B1H selection) [27] are also shown. Each nine bp target site was named as follows: “gene name or abbreviation”, ”exon number”, and “L” or “R” indicating left or right half-site. The amino acids selected in the three zinc finger recognition helices of each array are shown (residues are shown left to right in the order −1, 1, 2, 3, 4, 5, 6 numbered relative to the helix start). B2H values that fall below the cut-off of three-fold activation in the B2H system are italicized. The names of zinc finger arrays tested as ZFNs in zebrafish are shown in bold italics. Abbreviations key: DT = *dopamine transporter*, HIF = *hif1aa*, Telo = *telomerase*, TfR2 = *transferrin receptor 2*, and Grck = *gridlock*.

### Efficient somatic cell mutation of endogenous zebrafish genes using OPEN ZFNs

We next tested the abilities of zinc finger arrays obtained by OPEN to induce mutations when expressed as ZFNs in somatic zebrafish cells. To do this, we chose one zinc finger array for each ZFN target half-site and tested pairs as ZFNs (highlighted in bold italics in **Table 1**). To test the robustness of our OPEN selections, we chose zinc finger arrays with high (but not always the highest) B2H fold-activation for testing as ZFN pairs; however, all arrays tested met the minimum three-fold B2H activation threshold described above. DNA fragments encoding these zinc finger arrays were cloned into ZFN expression vectors previously constructed by the Joung lab (see Materials and Methods for details) [26]. The ten resulting vectors encode ZFNs consisting of a FLAG epitope tag, an SV40 nuclear localization signal, and a zinc finger array fused to an obligate heterodimeric *Fok*I nuclease domain [43]. These vectors also harbor a bacteriophage T7 promoter positioned upstream of the ZFN coding sequence. As a positive control, we also constructed two additional ZFN expression plasmids which encoded obligate heterodimeric ZFNs harboring the Wolfe/Lawson *kdr* zinc finger arrays (ZFP1 and ZFP2) [27]. We note that these control *kdr* ZFN plasmids are identical to our OPEN ZFN expression vectors except for the sequences encoding the zinc finger arrays. We transcribed RNA from each of these 12 ZFN expression plasmids and performed poly A-tailing of the RNA as described in Materials and Methods.

**Figure 2:**
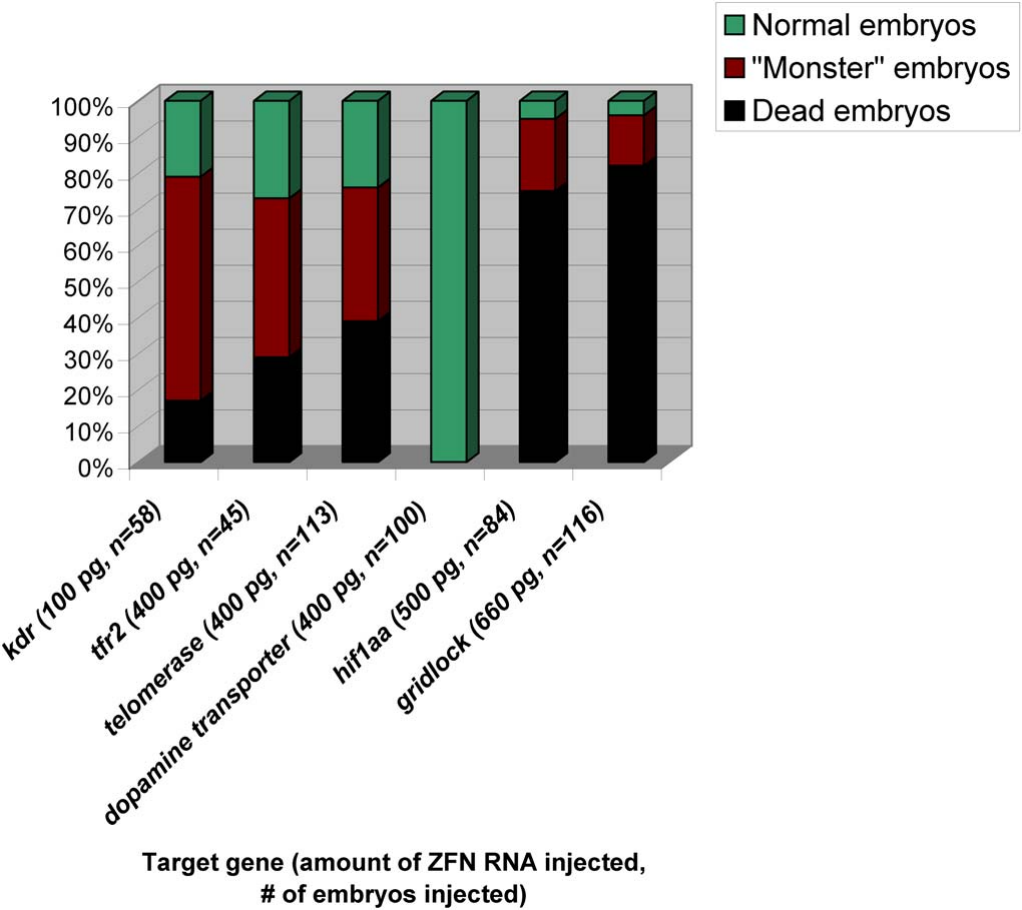
Toxicity and teratogenicity of OPEN and B1H-selected ZFNs in zebrafish embryos. Percentages of dead, deformed (“monster”), and normal embryos following injection with the amounts of ZFN RNAs indicated are shown. Percentages were calculated from the number of embryos (n) indicated.

**Figure 3:**
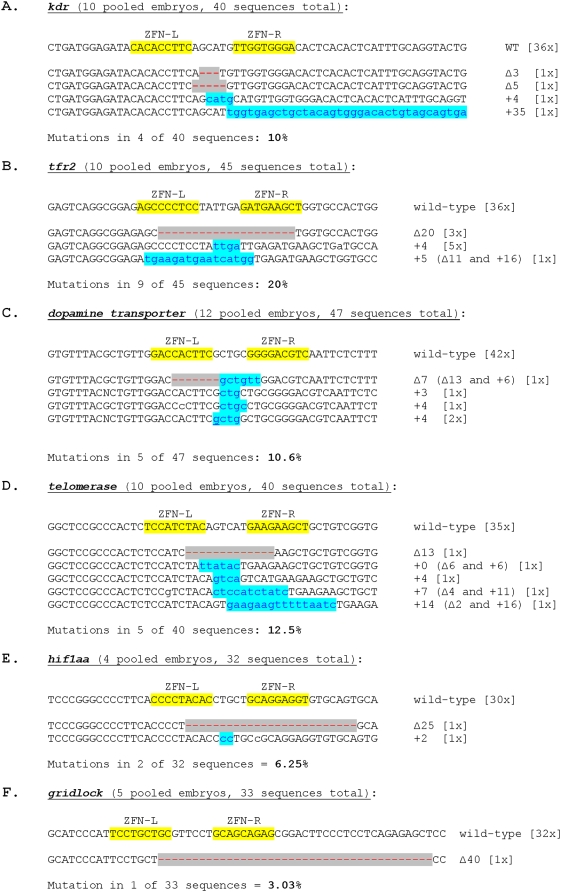
Frequencies and sequences of ZFN-induced mutations in somatic zebrafish cells. For each gene targeted by ZFNs, the wild-type sequence is shown at the top with ZFN binding sites marked. Deletions are indicated by grey highlighted red dashes and insertions by blue highlighted lower case blue letters. The number of times each wild-type mutant allele was isolated is shown in brackets.

In an initial control experiment to test whether our ZFN vectors and experimental conditions would work efficiently in zebrafish, we injected ∼100 embryos each with 100 pg of purified RNA made from the pair of vectors encoding the Wolfe/Lawson *kdr* ZFNs (50 pg of RNA encoding each ZFN). As shown in **Figure 2**, we observed that approximately 79% of the embryos were dead or exhibited a highly deformed “monster” phenotype, consistent with previously published experiments performed with these ZFNs [27]. Furthermore, we observed that we could not inject more than 100 pg of RNA/embryo without causing death in almost all embryos (data not shown). To assess whether targeted mutagenesis of *kdr* occurred at the somatic cell level, we harvested genomic DNA from a pool of 10 embryos two days post-injection and sequenced the region of the *kdr* gene targeted by the Wolfe/Lawson ZFNs using a limited cycle PCR/DNA-sequencing method previously described and validated by the Joung lab for quantitation of mutations in a population of alleles [26]. As shown in **Figure 3A**, 10% of the *kdr* alleles we sequenced harbored insertions or deletions at the site of the ZFN-induced DSB, a mutagenesis efficiency comparable to that previously observed by Wolfe and Lawson [27].

Having validated our ZFN expression vectors and mutation detection assay, we next tested each of the five ZFN pairs we made by OPEN in zebrafish embryos. Interestingly, with our OPEN ZFNs, we were able to inject four-times or more RNA per embryo (400 to 660 pg of RNA/embryo) compared with the Wolfe/Lawson *kdr* ZFNs and yet observed comparable or sometimes lower rates of death/monster formation (**Figure 2**). When OPEN ZFNs were injected at 100–200 pg of RNA/embryo, death/monster rates were even lower (0–27%; data not shown). To check for evidence of mutations in somatic cells, we isolated pooled genomic DNA from 4–10 embryos for each pair of ZFNs and performed limited cycle PCR/DNA sequencing to assess whether mutations were introduced at their intended endogenous gene targets. As shown in **Figures 3B–3F**, we observed insertion or deletions at the ZFN cleavage site for all five endogenous genes with mutagenesis rates ranging from 3%–20%. Nearly all of these indel mutations are predicted to create frameshift mutations although a few frame-preserved mutations are also observed. We conclude that OPEN ZFNs can efficiently induce mutations at endogenous genes in somatic zebrafish cells.

### Efficient germline transmission of mutations induced by OPEN ZFNs

**Figure 4:**
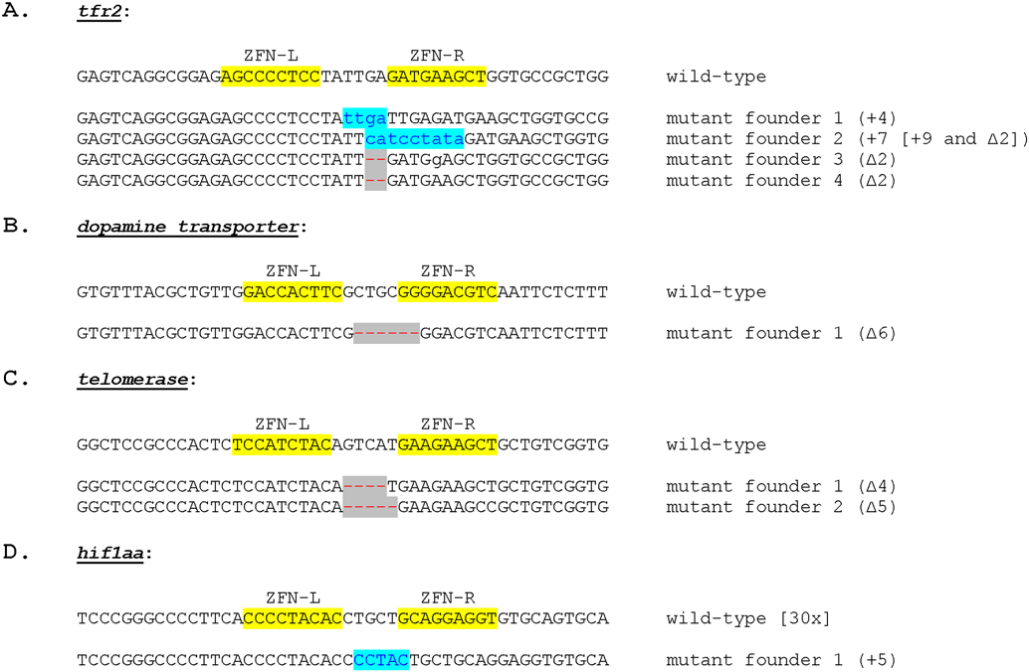
Sequences of ZFN-induced mutations transmitted through the germline. For each target gene, the wild-type sequence is shown at the top with ZFN binding sites marked and the mutated alleles found in founder progeny are shown below the wild-type sequence. Each mutant sequence shown was isolated from progeny of different founders. Deletions are indicated by grey highlighted red dashes and insertions by blue highlighted lower case blue letters.

We tested whether ZFN-induced mutations observed in somatic zebrafish cells could be transmitted efficiently through the germline. Injected embryos remaining from four of the five somatic cell experiments described above were allowed to mature to adulthood and crossed with wild-type fish (fish in which *gridlock* had been targeted have not yet reached maturity and therefore have not yet been tested). To identify founders, we analyzed individual embryos from these crosses using either direct DNA sequencing or a restriction digest assay that checks for the loss of a restriction site located at the ZFN-induced DSB (see Materials and Methods). As shown in **Table 2**, founders were identified at frequencies of ∼6%, 33%, 25%, and 50% for mutations in the *dat, tfr2, telomerase and hif1aa* genes, respectively. The percentages of embryos harboring ZFN-induced mutations from founders ranged from 9% to 60% (**Table 2**). We sequenced the mutations from a subset of these embryos to determine the molecular nature of the indels and found both frame-shifted and frame-preserved mutations (**Figure 4**). We conclude that mutations generated by OPEN ZFNs undergo efficient germline transmission in zebrafish.

**Table 2:** Frequencies of mutations from founder analysis

Gene	# of fish screened	# of mutant founder fish	transmission rate for each mutant founder			
***tfr2***	12	4	1/10 (10%)	1/10 (10%)	2/11 (18%)	6/10 (60%)
***dat***	17	1	6/30 (20%)			
***telo***	8	2	1/11 (9%)	1/11 (9%)		
***hif1 aa***	2	1	1/11 (9%)			

Numbers of fish screened for mutant founders are shown for each gene. For each mutant founder identified, the transmission rate is shown as the # of mutant embryos/# of embryos screened and as a percentage of mutant embryos identified (in parentheses). *tfr2 = transferrin receptor 2; dat = dopamine transporter; telo = telomerase; hif1aa = hypoxia-inducible factor.*

### *In silico* identification of OPEN ZFN targets within endogenous zebrafish genes

Using a program similar to the web-based software program ZiFiT v3.0 [26], [44], we searched for sites in endogenous zebrafish genes that could potentially be targeted by OPEN (see Materials and Methods). To do this, we searched the first three coding exons of 29,291 protein-coding gene transcripts that have known mapped chromosomal locations and that are present in the Ensembl *Danio rerio* database (release 51; http://www.ensembl.org/Danio_rerio/Info/Index). In total, we found 315,186 potential ZFN sites in these 29,291 gene transcripts (**Supplemental Tables S1, S2, S3, S4, S5, S6, S7, S8, S9, S10, S11, S12, S13, S14, S15, S16, S17, S18, S19, S20, S21, S22, S23, S24, S25)**. Importantly, we identified one or more potential OPEN ZFN sites (mean of ∼4.5 sites) in the first three coding exons of 25,174 of these gene transcripts and two or more potential OPEN ZFN sites (mean of ∼5.4 sites) in the first three-coding exons of 20,418 gene transcripts (**Table 3**). These results suggest that OPEN could potentially be used to generate ZFNs for as many as 86% of the transcripts encoded in the zebrafish genome.

**Table 3:** Summary of potential OPEN ZFN target sites identified in zebrafish transcripts

Number of transcripts with:	…in the first coding exon	…in the first two coding exons	…in the first three coding exons
**One or more potential ZFN sites…**	14,623	21,781	25,174
**Two or more potential ZFN sites…**	9,292	15,961	20,418

A total of 29,291 gene transcripts derived from the Ensembl release 51 *Danio rerio* database were analyzed for potential OPEN ZFN target sites (see Materials and Methods for additional details).

## Discussion

In this report, we used the recently described OPEN zinc finger engineering method to rapidly generate ZFNs that can modify endogenous zebrafish gene targets with high efficiency. In less than two months time, we generated ZFNs for target sites in five different biologically important genes. The five pairs of ZFNs we tested can efficiently generate mutations at their intended endogenous gene target in somatic zebrafish cells. In addition, we demonstrated that mutations induced by OPEN ZFNs can be efficiently transmitted through the germline. These results suggest that only a small number of fish need to be screened to identify founders, consistent with previously published results [27], [28]. Our results nearly triple the total published number of endogenous zebrafish genes successfully modified using ZFNs from three (*kdr*, *golden*, and *ntl*) to eight and demonstrate that OPEN is a highly effective ZFN engineering method for creating targeted mutations in zebrafish.

*In silico* analysis indicates that as many as 25,174 zebrafish gene transcripts have one or more potential OPEN ZFN target sites in their first three coding exons and as many as 20,418 transcripts have two or more sites in the first three coding exons. Our previous experience using OPEN to make ZFNs for endogenous human gene targets suggests that the success rate of this method is high but not 100% [26]. Assuming that a similar experience holds true in zebrafish, a prudent strategy might be to target more than one sequence in a gene of interest to improve the chances of successfully mutating that gene. Our analysis shows that most zebrafish gene transcripts possess multiple potential OPEN ZFN target sites and therefore suggests that a large number of genes will be targetable by such a strategy. As the academic community collectively gains experience using OPEN ZFNs in zebrafish (and other organisms), we anticipate that algorithms for identifying potential OPEN ZFN target sites will also continue to improve and evolve, thereby allowing for targeting of fewer sites per gene. Nonetheless, our results strongly suggest that OPEN in its current form can be used to engineer ZFNs for a high percentage of zebrafish genes.

The reagents we used to construct and express ZFNs in our experiments are part of the OPEN Zinc Finger Consortium platform for engineering zinc finger arrays. Zinc finger arrays constructed by OPEN can be excised as *Xba*I/*BamH*I fragments and cloned directly into expression plasmids which then encode FLAG-tagged, NLS-tagged, obligate heterodimeric ZFNs. ZFN-encoding RNA can be directly transcribed from these plasmids using T7 RNA polymerase. OPEN Zinc Finger Consortium reagents are publicly available to academic laboratories through either Addgene (a non-profit plasmid distribution service; see http://www.addgene.org/zfc) or the Joung lab. All engineering and validation steps in the OPEN method are carried out in simple and inexpensive *E. coli*-based systems. Collectively, these reagents provide academics with an important platform which enables rapid engineering of ZFNs for zebrafish genes.

When compared with ZFNs previously made to the *kdr* gene using a bacterial one-hybrid-based method, OPEN ZFNs were equally efficient at inducing mutations at their respective target sites but showed less toxicity and teratogenesis, even when larger amounts of ZFN-encoding RNA were injected. (Others have also noted the relatively greater toxicity of the *kdr* ZFNs compared with the *golden* and *ntl* ZFNs made using the Sangamo BioSciences zinc finger engineering technology [29], [45] although we note that those comparisons were made between experiments which used different ZFN expression vectors.) The difference in toxicity we observed between the *kdr* ZFNs and our OPEN ZFNs is most likely due to the quality of the zinc finger arrays because all other sequences in the expression vectors are otherwise identical. One possible explanation for the greater toxicity of the *kdr* ZFNs is that the B1H system used to create them might permit identification of zinc finger arrays with lower specificities than those identified by the OPEN B2H system. Consistent with this, we note that one of the zinc finger arrays (ZFP2) used to make the *kdr* ZFNs failed to activate transcription in the B2H system (**Table 1**) and therefore would not have been identified as a positive clone if the *kdr* site had been targeted using the OPEN method. This result suggests that the B1H method used to engineer the *kdr* zinc finger arrays may be less stringent than the B2H-based OPEN approach, perhaps due to the use of a multi-copy reporter in the B1H system as opposed to the single-copy reporter used in the B2H system. This difference might reduce the selective pressure for DNA-binding specificity since the target DNA site in the B1H system will be present at a higher concentration in the bacterial cell relative to the “non-specific” DNA of the *E. coli* chromosome. An important priority for future work will be to examine the spectrum and range of “off-target” sites altered by OPEN ZFNs, perhaps using methods previously described by other groups [27], [28].

The ability of OPEN to rapidly yield high quality ZFNs for a large number of different target sites should also improve prospects for using ZFNs to induce precise homologous recombination (**HR**) events at endogenous zebrafish genes. Repair of a ZFN-induced DSB by HR with an appropriately designed exogenous “donor template” (a process known as ZFN-induced gene targeting) has been used to introduce specific alterations or insertions with high efficiencies at endogenous genes in *Drosophila* [18], [19], plant [Townsend et al, *manuscript submitted*], and human cells [23]–26, 43. However, our experience using ZFNs in human cells suggests that ZFN-induced homologous recombination can be much more challenging to implement than ZFN-induced mutagenic NHEJ-mediated repair. For example, not all ZFNs that can induce NHEJ at their target sites will necessarily promote efficient HR (M. Maeder, S. Beganny, and J.K. Joung, unpublished results). Thus, the ability to use the OPEN method to rapidly engineer ZFNs with both high activities and low toxicities for many different target sites should greatly enhance prospects for successfully using ZFNs to induce specific HR events in zebrafish.

## Materials and Methods

### OPEN selections of zinc finger arrays

Zinc finger arrays were selected using the OPEN method essentially as previously described [26] but with a small number of alterations that improve the speed and throughput of the protocol. We briefly summarize the overall protocol here with greater detail provided for steps of the protocol we altered for this report. A more detailed step-by-step protocol for performing OPEN selections is forthcoming (Maeder et al., manuscript in preparation) and is currently available upon request from the Joung lab.

To create recombinant libraries of zinc finger arrays for use in OPEN selections, zinc finger pools for target triplet subsites [26] were amplified by PCR using primers and conditions as previously described [26]. Amplified finger pool products were purified on 10% polyacrylamide gels and then fused together by PCR to create random combinations of three-finger arrays. These fusion PCR reactions were performed with equal concentrations of the three purified finger pool fragments and using primers and cycling conditions as previously described [26]. The resulting PCR product encoding a collection of three-finger arrays was purified on a 5% polyacrylamide gel and treated with *Pfu* polymerase and T4 polynucleotide kinase to create ligation-ready overhangs [26]. This fragment was then ligated to pBR-UV5-GP-FD2 vector that had been digested with the restriction enzyme *Bbs*I. The resulting plasmids express the collection of zinc finger arrays as FLAG-tagged Gal11P fusions in the B2H system. Electroporation was then used to introduce these ligation products into *E. coli* XL-1 Blue cells and >3×10^6^ independent transformants were obtained for each library to ensure a minimum of three-fold oversampling of the theoretical library complexity of ∼8.6×10^5^ (95^3^). Libraries were then converted into infectious M13 phage as previously described [46].

OPEN selections were performed in two stages. In the first stage, an OPEN three-finger library was introduced by infection into a B2H selection strain harboring the full target DNA sequence of interest. For the selections described in this report, we grew selection strains as 1 ml cultures in 24-well, 10 ml-capacity pyramidal well blocks in a Microtitertron shaker (Appropriate Technical Resources, Inc.) at 350 rpm, 37°C, 80% humidity. These cultures were grown in NM medium supplemented with 30 μg/ml chloramphenicol, 30 μg/ml kanamycin, and 50 μM IPTG. Following overnight growth, selection strain cultures were infected with a matched combinatorial zinc finger array phagemid phage library constructed as described above. Following phage infection, 4 ml of NM medium [46] supplemented with 30 μg/ml chloramphenicol, 30 μg/ml kanamycin, and 50 μM IPTG was added to the cells which were then shaken on the Microtitertron shaker for 1.5 hrs. The infected cells were then spun down and 4ml of the supernatant removed. The cell pellet was then resuspended in the remaining 1 ml of liquid media and 250 μl of this resuspension was plated on two different NM/CCK medium plates containing 50 μM IPTG, 10 mM 3AT, and 20 μg/mL streptomycin or 50 μM IPTG, 25 mM 3AT, and 40 μg/mL streptomycin. After 36–48 hours of incubation, colonies were harvested from the highest stringency plate yielding at least 1000 colonies as previously described [26]. The resulting cell suspension was then diluted with 4.5 ml 2XYT media supplemented with 50 μg/ml carbenicillin and 30 μg/ml kanamycin to an OD_600_≈0.1 in the 10 ml-capacity well of a 24-well block and allowed to grow for 1 hour in the Microtitertron shaker as described above. This subculture was infected with 10^11^ kanamycin transducing units of M13K07 helper phage and then grown for six hours in the Microtitertron shaker. Phage-containing culture supernatants were harvested by filtering the cell cultures through a 0.22 μm polyethersulfone syringe filter.

In the second stage of OPEN selection, selection strain cells were again grown in 24-well blocks but in 1 ml of NM medium supplemented with 30 μg/ml chloramphenicol, 30 μg/ml kanamycin, and no IPTG. This overnight culture was infected with ∼6×10^5^ ampicillin-transducing units (ATUs) of zinc finger-encoding phagemid phage rescued from the initial stage of selection. Following infection, 400 μl of NM medium supplemented with 30 μg/ml chloramphenicol and 30 μg/ml kanamycin was added to the cells which were then shaken on the Microtitertron shaker for 1.5 hrs. 375 μl of this infected culture (corresponding to ∼5×10^−5^ infected/transformed cells) was then plated on a square 100mm×100mm NM medium agar plate supplemented with 100 μg/ml carbenicillin, 30 μg/ml chloramphenicol, 30 μg/ml kanamycin and containing gradients of 3-aminotriazole (from 0 to 80 mM) and streptomycin (from 0 to 100 μg/ml). Gradient plates were poured as previously described [26].

### Construction of ZFN expression vectors

DNA sequences encoding zinc finger arrays identified by OPEN were transferred to ZFN expression vectors by using the phagemids encoding these arrays as templates for PCR reactions using primers OK.1677 and OK.1678 (**Supplemental Table 26**). The resulting DNA fragments (encoding the zinc finger arrays) were digested with *Xba*I and *BamH*I and cloned into *Xba*I/*BamH*I-digested ZFN expression vectors pMLM335 or pMLM336 [26]. The pMLM335 and pMLM336 vectors encode previously described obligate heterodimeric ZFNs [43]. Final sequence-verified plasmids were prepared using a QIAgen HiSpeed Midiprep kit using RNase free reagents and stored in RNase-free Eppendorf Safe Lock Tubes.

### Preparation of ZFN-encoding RNA

ZFN expression vectors were linearized with *Pme*I (an enzyme which cleaves just 3′ to the end of the ZFN coding sequence) and transcribed *in vitro* using the T7 mMessage mMachine kit (Ambion). The transcribed ZFN RNAs were then polyadenylated using the Poly(A) Tailing kit (Ambion).

### Injection of zebrafish and analysis of somatic mutations

Approximately 2 nl of the ZFN RNA (at concentrations of 50–400 pg/nl) was injected into one-cell stage zebrafish embryos. Two days following fertilization, the surviving injected embryos were grouped into either “normal” or “deformed” phenotypes. Genomic DNA was extracted from pools of 4–12 embryos from each “normal” group using DNA extraction buffer (10 mM Tris, pH 8.0, 200 mM NaCl, 10 mM EDTA, 0.5% SDS, 100 μg/ml Proteinase K), followed by phenol/chloroform extraction and ethanol precipitation. The DNA was resuspended in 40 μl of TE (10 mM Tris, pH 8.0, 1 mM EDTA).

2.5 μl of the resulting genomic DNA was then used as template for a PCR reaction using Platinum Taq DNA Polymerase High Fidelity enzyme (Invitrogen) with primers designed to anneal approximately 150 to 200 bp upstream and downstream from the expected mutation. The resulting PCR product was cleaned up using a QIAGEN Minelute PCR purification kit and then ligated using a ZeroBlunt TOPO kit (Invitrogen) into linearized pCR4 Blunt-TOPO vector. The ligation was transformed into Mach1 T1-bacteriophage resistant *E.coli* (Invitrogen) and plated on LB plates containing 50 μg/ml kanamycin. Following incubation overnight at 37°C, colonies were picked from these plates and inoculated into 700 μl TB medium containing 50 μg/ml kanamycin in 96-well blocks with 1ml pyramidal-bottom wells. These blocks were shaken at 900 rpm, 37°C, and 80% humidity in a Microtitertron shaker. Plasmid DNA was isolated from these cultures and sent for sequencing using the “T3 sequencing” primer (**Supplemental Table 26**).

### Identification and sequencing of germline transmitted mutations

Potential founders were crossed with wild-type zebrafish. One to three dpf (days post fertilization), progeny were lysed individually in lysis buffer (10 mM Tris, pH 8.0, 2 mM EDTA, 0.1% Triton X-100, 100 μg/ml Proteinase K) and incubated at 50°C overnight. For each target gene, 10–12 embryos from each potential founder were screened for the presence of ZFN-induced mutations by amplifying the region surrounding the relevant ZFN cleavage site by PCR and then using either restriction digest- and/or DNA sequencing-based assays: For the *tfr2* gene, we used primers OK.1922 and OK.1923 to amplify the region surrounding the ZFN target site by PCR and the resulting ∼405 bp product from each embryo was directly sequenced with primer Tfr2-seq (**Supplemental Table 26**). For the *dopamine transporter* gene, we used primers OK.1916 and OK.1917 (**Supplemental Table 26**) to amplify the region surrounding the ZFN target site by PCR and the resulting ∼418 bp product was digested with the restriction enzyme *ApeK*I. The PCR product from a wild-type allele will yield 5 fragments of 251, 68, 52, 25 and 7-bp sizes. Introduction of indel mutations at the ZFN target site will cause disruption of the *ApeK*I site and result in the appearance of an additional 120-bp fragment which is detectable on a 3% agarose gel. PCR fragments from selected progeny that showed evidence for loss of the *ApeK*I site were blunt-end cloned into the pCR4 Blunt-TOPO vector as described above and sequenced with the “T3 sequencing” primer. For the *telomerase* gene, we used primers OK.1928 and OK.1930 (**Supplemental Table 26**) to amplify the surrounding region by PCR and the resulting ∼306 bp product was digested with the enzyme *BspH*I. The PCR product from a wild-type allele will contain only one *BspH*I site. Introduction of indel mutations at the ZFN target site will disrupt the *BspH*I site thereby resulting in the generation of PCR products resistant to digestion by *BspH*I. PCR fragments from progeny that were resistant to *BspH*I were blunt-end cloned into the pCR4 Blunt-TOPO vector as described above and sequenced with the “T3 sequencing” primer. For the *hifaa* gene, we used primers OK.1934 and OK.1935 (**Supplemental Table 26**) to amplify the surrounding region by PCR and the resulting ∼401 bp product was digested with the enzyme *BfuA*I. The PCR product from a wild-type allele will contain only one *BfuA*I site. Introduction of indel mutations at the ZFN target site will disrupt the *BfuA*I site thereby resulting in the generation of PCR products resistant to digestion by *BfuA*I. PCR fragments from progeny that were resistant to *BfuA*I were blunt-end cloned into the pCR4 Blunt-TOPO vector as described above and sequenced with the “T3 sequencing” primer.

### Identification of potential OPEN ZFN target sites in zebrafish transcripts

ZFN target sites were generated from *Danio rerio* chromosomal contigs (Zv7) and gene table files (updated July 2008) from Ensemble (http://www.ensembl.org) for all mapped chromosomal protein coding transcripts. ZFN target sites were identified that can be targeted using currently available OPEN reagents [26] and that possess a spacer of 5, 6, or 7 nucleotides between the target half-sites. Only ZFN sites whose spacer falls entirely within an exon were identified as potential targets. In addition, because all previous 9 bp sites successfully targeted by OPEN to date have possessed at least one GNN triplet [26], we eliminated ZFN sites harboring one or more half-sites that are devoid of GNN triplets. Finally, because OPEN selections are performed in *E. coli*, ZFN sites containing either a *dam* or a *dcm* methylation site in either half-site were also eliminated from the target list.

## Supporting Information

Table S1 Potential OPEN ZFN target sites in gene transcripts encoded on zebrafish chromosome 1. Potential OPEN ZFN target sites within transcripts were identified as described in Materials and Methods. Gene ID and Transcript ID are from the Ensembl *Danio rerio* release 51 database. “Strand” indicates whether the “Target Site” shown (written 5′ to 3′) occurs on the forward (+) or reverse (−) strand. “ZFN Spacer Length” indicates the length of the spacer sequence located between the ZFN half-sites (5, 6, or 7 bps). “Coding Sequence Length” indicates the total nucleotide length of the coding sequence within the transcript and “ZFN Cleavage Site” indicates the nucleotide position of the cleavage site (i.e.–the first base of the “Target Site”) within the coding sequence.
(2.22 MB XLS)

Table S2 Potential OPEN ZFN target sites in gene transcripts encoded on zebrafish chromosome 2. Data presented as described in the legend to Table S1.
(2.39 MB XLS)

Table S3 Potential OPEN ZFN target sites in gene transcripts encoded on zebrafish chromosome 3. Data presented as described in the legend to Table S1.
(2.28 MB XLS)

Table S4 Potential OPEN ZFN target sites in gene transcripts encoded on zebrafish chromosome 4. Data presented as described in the legend to Table S1.
(2.05 MB XLS)

Table S5 Potential OPEN ZFN target sites in gene transcripts encoded on zebrafish chromosome 5. Data presented as described in the legend to Table S1.
(3.12 MB XLS)

Table S6 Potential OPEN ZFN target sites in gene transcripts encoded on zebrafish chromosome 6. Data presented as described in the legend to Table S1.
(2.28 MB XLS)

Table S7 Potential OPEN ZFN target sites in gene transcripts encoded on zebrafish chromosome 7. Data presented as described in the legend to Table S1.
(2.80 MB XLS)

Table S8 Potential OPEN ZFN target sites in gene transcripts encoded on zebrafish chromosome 8. Data presented as described in the legend to Table S1.
(2.44 MB XLS)

Table S9 Potential OPEN ZFN target sites in gene transcripts encoded on zebrafish chromosome 9. Data presented as described in the legend to Table S1.
(2.21 MB XLS)

Table S10 Potential OPEN ZFN target sites in gene transcripts encoded on zebrafish chromosome 10. Data presented as described in the legend to Table S1.
(1.78 MB XLS)

Table S11 Potential OPEN ZFN target sites in gene transcripts encoded on zebrafish chromosome 11. Data presented as described in the legend to Table S1.
(1.75 MB XLS)

Table S12 Potential OPEN ZFN target sites in gene transcripts encoded on zebrafish chromosome 12. Data presented as described in the legend to Table S1.
(1.55 MB XLS)

Table S13 Potential OPEN ZFN target sites in gene transcripts encoded on zebrafish chromosome 13. Data presented as described in the legend to Table S1.
(1.98 MB XLS)

Table S14 Potential OPEN ZFN target sites in gene transcripts encoded on zebrafish chromosome 14. Data presented as described in the legend to Table S1.
(1.62 MB XLS)

Table S15 Potential OPEN ZFN target sites in gene transcripts encoded on zebrafish chromosome 15. Data presented as described in the legend to Table S1.
(1.84 MB XLS)

Table S16 Potential OPEN ZFN target sites in gene transcripts encoded on zebrafish chromosome 16. Data presented as described in the legend to Table S1.
(1.93 MB XLS)

Table S17 Potential OPEN ZFN target sites in gene transcripts encoded on zebrafish chromosome 17. Data presented as described in the legend to Table S1.
(2.02 MB XLS)

Table S18 Potential OPEN ZFN target sites in gene transcripts encoded on zebrafish chromosome 18. Data presented as described in the legend to Table S1.
(2.13 MB XLS)

Table S19 Potential OPEN ZFN target sites in gene transcripts encoded on zebrafish chromosome 19. Data presented as described in the legend to Table S1.
(2.06 MB XLS)

Table S20 Potential OPEN ZFN target sites in gene transcripts encoded on zebrafish chromosome 20. Data presented as described in the legend to Table S1.
(2.79 MB XLS)

Table S21 Potential OPEN ZFN target sites in gene transcripts encoded on zebrafish chromosome 21. Data presented as described in the legend to Table S1.
(1.72 MB XLS)

Table S22 Potential OPEN ZFN target sites in gene transcripts encoded on zebrafish chromosome 22. Data presented as described in the legend to Table S1.
(2.11 MB XLS)

Table S23 Potential OPEN ZFN target sites in gene transcripts encoded on zebrafish chromosome 23. Data presented as described in the legend to Table S1.
(1.98 MB XLS)

Table S24 Potential OPEN ZFN target sites in gene transcripts encoded on zebrafish chromosome 24. Data presented as described in the legend to Table S1.
(1.46 MB XLS)

Table S25 Potential OPEN ZFN target sites in gene transcripts encoded on zebrafish chromosome 25. Data presented as described in the legend to Table S1.
(1.58 MB XLS)

Table S26 Sequences of primers used in this study
(0.02 MB XLS)
